# Konzo: From Poverty, Cassava, and Cyanogen Intake to Toxico-Nutritional Neurological Disease

**DOI:** 10.1371/journal.pntd.0001051

**Published:** 2011-06-28

**Authors:** Hipólito Nzwalo, Julie Cliff

**Affiliations:** 1 Faro Central Hospital, Faro, Portugal; 2 Department of Community Health, Faculty of Medicine, Universidade Eduardo Mondlane, Maputo, Mozambique; London School of Hygiene & Tropical Medicine, United Kingdom

## Abstract

Konzo is a distinct neurological entity with selective upper motor neuron damage, characterized by an abrupt onset of an irreversible, non-progressive, and symmetrical spastic para/tetraparesis. Despite its severity, konzo remains a neglected disease. The disease is associated with high dietary cyanogen consumption from insufficiently processed roots of bitter cassava combined with a protein-deficient diet. Epidemics occur when these conditions coincide at times of severe food shortage. Up to 1993, outbreaks in poor rural areas in Africa contributed to more than 3,700 cases of konzo. The number of affected people is underestimated. From unofficial reports, the number of cases was estimated to be at least 100,000 in 2000, in contrast to the 6,788 cases reported up to 2009 from published papers.

## Introduction

Konzo is a distinct neurological entity with selective upper motor neuron damage, characterized by an abrupt onset of an irreversible, non-progressive, and symmetrical spastic para/tetraparesis [1]–[8].

The disease is associated with prolonged high dietary cyanogen consumption from insufficiently processed roots of bitter cassava combined with a protein-deficient diet low in sulphur amino acids (SAAs) [1]–[8].

Since its first description by the Italian doctor Trolli eight decades ago in the former Belgian Congo (now the Democratic Republic of Congo [DRC]), epidemics have been reported from many cassava-consuming areas in rural Africa. Up to 1993, the total of reported cases was approximately 3,700 to 4,000 [9]–[11].

**Figure 1:**
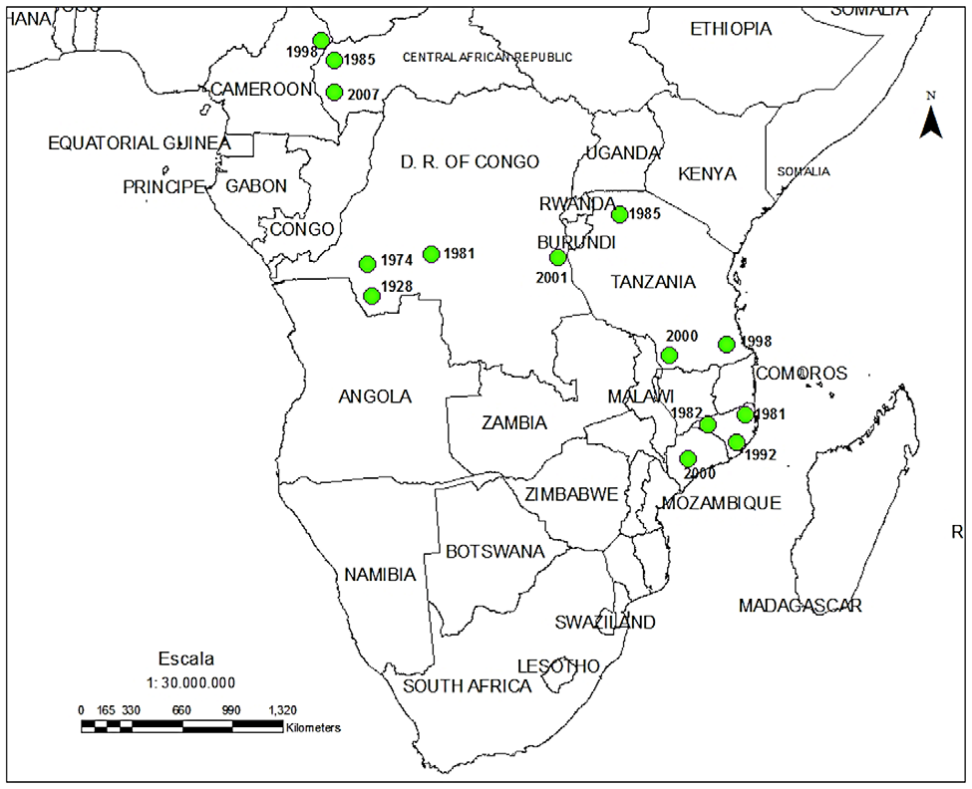
Countries in Africa where konzo has been reported.

Konzo remains a health problem in Africa. Since 1993, the disease has extended beyond its first reported boundaries [12], and the reported number of konzo cases has almost doubled, reaching a total of 6,788 (Table 1, Figure 1).

**Table 1:** Total number of konzo cases reported up to 2009.

Country	Prior to 1975	1975–1993	1994–2009	Total
Democratic Rebublic of Congo	1,237	919	1,303	3,459
Mozambique		2,123	281	2404
Tanzania		121	238	359
Central African Republic		16	81	97
Cameroon			469	469
Total	1,237	3,179	2,372	6,788

Adapted from [9].

Outbreaks in past decades in Cameroon [12], Mozambique [13], [14], Tanzania [15], the Central African Republic [16], and the DRC [17], [18] often received insignificant attention from the media and local health authorities despite the clinical severity of konzo. As in the earlier reported outbreaks, poverty in association with agricultural crises provoked by drought or war was a constant feature. Those affected belonged to the poorest segments of the most remote rural areas of Africa, perpetuating the silence around the disease.

Cassava is also an important source of food in the tropics outside of Africa [19], and, although remote, the possibility of konzo occurring in these areas should be considered. Another disease associated with chronic cassava consumption, tropical ataxic neuropathy (TAN), has been described recently in India [20]. The same socioeconomic factors implicated in konzo were present, and in some cases, patients had clinical features compatible with konzo.

Lessons from the previous outbreaks indicate that the number of people affected by konzo is underestimated. Unofficial reports point to an alarming number of 100,000 cases in the DRC in 2000 [21].

Examples from the past confirm that the disease can be easily overlooked. In the 1986 Zaire (now DRC) epidemic, the actual number of cases was estimated to be at least twice as many as those reported [22]. In Mozambique, in 1981, during a severe drought, an outbreak of more than 1,000 cases of konzo occurred, and was associated with an almost exclusively cassava-based diet. The disease, locally named “Mantakassa”, was restricted to the remotest areas of the isolated northern province of Nampula. The extent of the epidemic was only revealed after an extensive 2-month active case detection exercise [23]. Nonetheless, cases were missed, and further research carried out by the authors found that the epidemic extended beyond its previously reported boundaries [14].

Cultural and religious aspects may also play a role in underreporting, particularly in sporadic cases of konzo. In the Congo, “konzo” was the name of a fetish used with traps to catch wild animals by weakening their legs and preventing their escape. In man, the appearance of konzo was interpreted as a kind of divine punishment or sorcery caused by improperly using the fetish [6].

Sporadic cases of konzo can be misdiagnosed if health care staff are not trained to recognize the symptoms. In 1981, in Mozambique, the disease was initially considered to be of viral origin [23]. In the DRC, a community-based survey revealed that cases of acute paraparesis initially attributed to poliomyelitis were in fact cases of konzo in the context of an outbreak [17].

The possibility of an infectious agent was considered in the early reported epidemics, particularly HTLV-I-associated myelopathy/tropical spastic paraparesis (HAM/TSP). Cerebrospinal fluid studies were, however, normal, and serology for the retroviruses (HIV or HTLV-1) implicated in progressive spastic myelopathy was negative [2], [8], [23]–[25].

In an era of climate transformation and continued insecurity, it is important to keep in mind the possibility of new epidemics of konzo in vulnerable areas, particularly in Africa. We therefore review the disease, from aetiology to clinical evolution.

## Methods

We aimed to provide a non-systematic review of konzo, using published and unpublished sources. For published literature, we first searched the Medline database using the search terms “konzo and cassava”, “konzo and cyanogen”, “konzo and cyanide”, and “spastic paraparesis and cassava”. This search yielded 52 articles. We then searched the Agricola database using the search term konzo, and obtained 15 articles. We reviewed all these published articles, three PhD theses on konzo, and a further 25 articles identified by reference review.

## From Poverty to Cyanogen Intoxication

### Monotonous Diet Based on Cassava

Cassava (*Manihot esculenta* Crantz) is a perennial 1–3 meter high tropical shrub. The leaves have a high content of protein and vitamins, and normally they are consumed after processing, which removes cyanogens. The major harvested organ is the root. The roots have a high content of carbohydrate and also small amounts of some vitamins and minerals. Their protein content is low and deficient in SAAs such as cystine and methionine [26]–[28].

From 1965 to 2000, cassava cultivation in Africa showed an extraordinary increase, from 35 million to 90 million tons, at least partly in response to declining soil fertility and increased cost of inorganic fertilizers. For countries such as DRC, Tanzania, and northern Mozambique, cassava is the most important crop for the largest proportion of farming households [29]–[31]. The amount of labour required for cassava cultivation is considerably less than that for other crops, and this is a major reason for its promotion and increasing use in HIV/AIDS-affected communities [32].

Cassava is drought tolerant, grows on poor soils without fertilizer where no other staple can be cultivated, and generates acceptable yields even on depleted and marginal lands. Its roots may be kept in the soil for extended time periods, securing a carbohydrate source in years of agricultural crisis in poor communities, and bridging the seasonal food gap during the hungry and dry season when other crops usually fail [31], [33].

It is no surprise that in times of agricultural crisis, cassava becomes the dominant, and sometimes the only, source of food.

### Cassava and Cyanogen Consumption

Many species of plants liberate hydrogen cyanide from cyanogenic glycosides (CGs), a phenomenon called cyanogenesis, as a defense mechanism against animals and marauding insects [34], [35]. Cassava is by far the most important human food source that uses cyanide as a defense mechanism [35].

Roots and leaves of cassava of all varieties contain CGs, mainly as linamarin, but also as lotaustralin, in different concentrations in their cellular vacuoles [34], [36].

**Figure 2:**
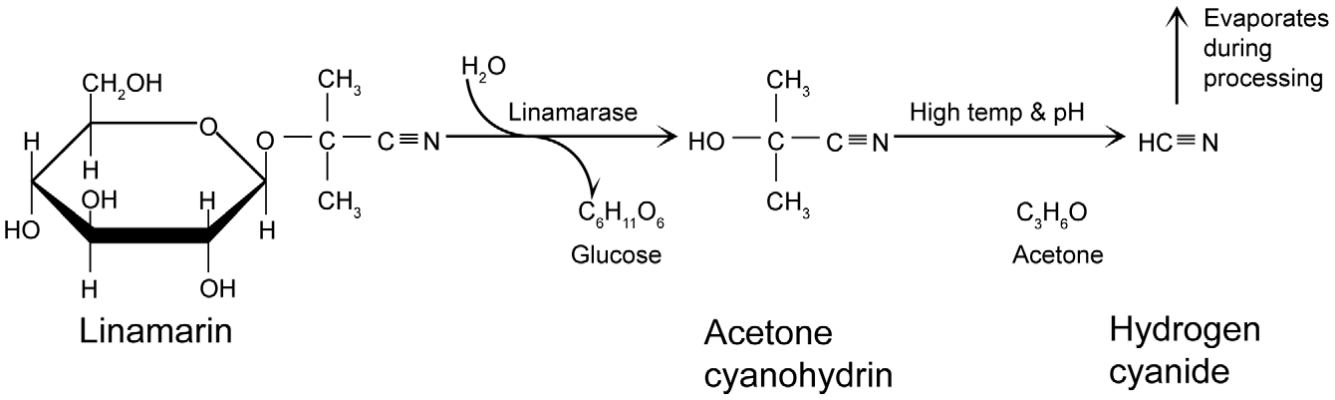
The cyanogenesis reaction.

Cyanogenesis is initiated in cassava when the plant tissue is damaged. Linamarase, a cell wall enzyme, is necessary for production of acetone cyanohydrin (AC) from the hydrolysis of linamarin (Figure 2). AC in cassava flour is unstable and can decompose to acetone and hydrogen cyanide spontaneously at pH>5.0 or at elevated temperatures (above 35°C), or enzymatically due to the action of hydroxynitrile lyase [34]–[37]. In gari, a commonly consumed cassava product, AC is quite stable at pH 4.2 at 50°C, and even at 100°C it is only slowly removed, and can only be removed if pH is raised to around 5 [38].

The concentration of CGs depends on both genetic and environmental factors. Water stress increases CG concentration, and agro-ecological differences can influence the cyanogenic potential of the same cassava cultivar [39], [40].

CGs in cassava can be reduced by appropriate processing of the plant material prior to consumption. Processing also improves palatability and increases shelf life, as the root suffers rapid post harvest deterioration if preserved in the fresh state for more than a few days [41], [42]. The final product may be flour (tapioca) or granules (gari).

High dietary cyanogen exposure occurs when high cyanogenic cassava and insufficient cassava processing are combined, usually in a context of food shortage.

In konzo-affected areas, insufficient cassava processing is attributed to short cuts in the established methods and is related to food shortage due to drought, crop failure, and sometimes commercialization of cassava [9]. Water stress may also increase the CG concentration in cassava to a level where the traditional processing methods can no longer avoid high retention of cyanogens [43], [44].

Cassava roots are processed by a variety of methods, depending on factors such as traditional preferences, time taken, and the availability of water and technology. Common methods used in Africa include soaking, sun drying, heap fermentation, and grating plus roasting [30], [42]. Mechanical disruption of plant cells during these processes allows a cyanogenesis reaction (Figure 2) to proceed, and thus CGs are eliminated in different degrees.

A new, feasible, and simple method, the “wetting method”, may be used for cassava flour, and decreases substantially the cyanide content [45]. This method has been successfully tested and accepted in rural communities in Mozambique and Tanzania, where water is in short supply and sun drying and heap fermentation are common processing methods [46]–[48]. In the DRC, where soaking is the prevalent method [3], a trial recently showed that the wetting method was acceptable, but the final results are still to be evaluated [48].

**Figure 3:**
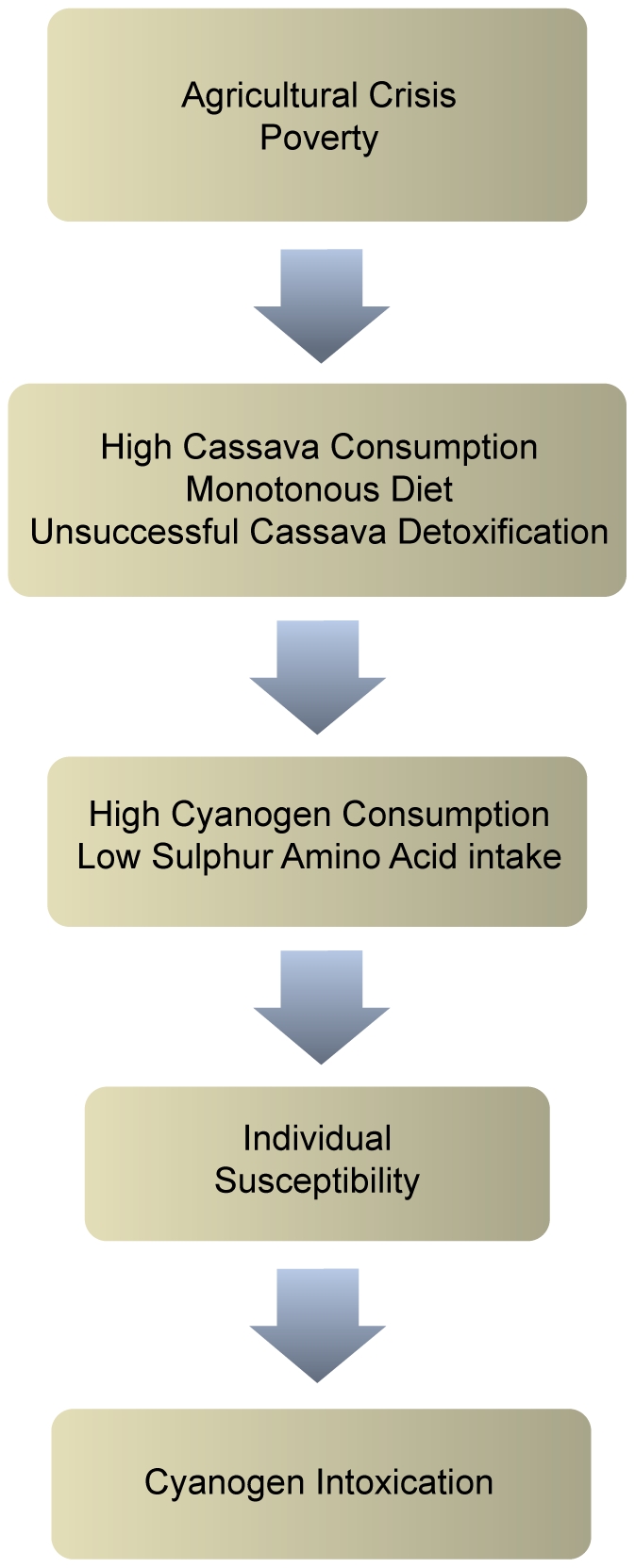
From poverty to cyanogen intoxication.

High cyanogen consumption is an endpoint of preventable factors acting in sequence or synergistically in the pre-pathogenic phase of konzo (Figure 3).

### Cyanogen Toxicity and Konzo

The association between the occurrence of konzo and high consumption of improperly processed cassava has been described.

In all major epidemics the appearance of konzo has been associated with a sustained and sub-lethal intake of cassava high in CG concentration in combination with a low SAA intake [1]–[8], [12]–[18].

The neurotoxic mechanism is not yet clarified and the existent neuropathological or imaging studies have not revealed any particular abnormality [4], [5], [49]. Neurophysiological studies in konzo point to involvement of either the corticomotorneurons or the descending motor pathways [5], [49].

Although several compounds have been proposed as candidates for the neurotoxic agent in konzo, few experimental studies involving those candidates have been reported. Table 2 summarizes the results of the principal experimental studies that have been carried out to date. The closest human model for konzo comes from primate experiments [55]. These have shown that the clinical and pathological findings in the central nervous system secondary to cyanate exposure are compatible with konzo.

**Table 2:** Experiments showing cyanogen neurotoxicity.

Subjects/Animals	Exposure	Relevant Neurotoxic Findings	Motor Changes and Similarities to Konzo
Neural phaeochromocytoma cell culture [50]	Linamarin (acute)	Direct linamarin-induced lesion neural culture	None
Rats on low SAA diet [51]	Linamarin (chronic)	Structural and functional proteomic modifications in the spinal cord	Non-motor symptoms; hind limb tremors can occur transiently at onset of konzo
Rats on low SAA diet [51]	Cyanate (chronic)	Structural and functional proteomic modifications in the spinal cord	Motor weakness, gait abnormalities resembling findings in konzo
Rats [52]	Cyanate (Acute)	Glutathione depletion by inhibition of glutathione reductase activity in the brain	None
Rats on low SAA diet [53]	Acetone cyanohydrin (chronic)	Structural brain lesions in nonmotor areas	None
Goats [54]	Cyanate (chronic)	Structural lesions at different levels of the nervous system (including ventral horn of the spinal cord and brainstem)	None
Rhesus monkeys [55]	Cyanate (chronic)	Structural lesions at different levels of the nervous system (Betz cells in the motor cortex , basal ganglia, and anterior horn cells)	Sudden onset of irreversible spastic quadriparesis resembling konzo, in association with general signs (wasting, anorexia)

SAA, sulphur amino acid.

Cyanide, a mitochondrial oxidative phosphorylation blocker [56], is the first described candidate for neurotoxicity in konzo [1]–[8]. There is an association at individual level between high blood cyanide and konzo [4], [57].

Cyanide comes from the conversion of linamarin, but we do not know where this happens most. Processing causes some conversion to AC, which probably decomposes to cyanide in the high pH of the gut [7], [9].

Cyanide is also absorbed through skin or the respiratory tract and is rapidly distributed throughout the body. Highest levels are typically found in the liver, lungs, blood, and brain [58].

Rhodanese is the enzyme responsible for most cyanide detoxification, transforming it to thiocyanate, which is subsequently excreted in the urine. The enzyme is located in all tissues, but mainly in the liver. This process requires sulphur donors, provided from dietary SAAs, which are deficient in cassava roots [58], [59].

The capture of cyanide with the iron in the erythrocytic haemoglobin to form methaemoglobin is an important temporary measure to keep the cyanide under control. Other mechanisms, such as capture with hydroxycobalamin (vitamin B12a) to yield cyanocobalamin (vitamin B12), provide no practical contribution to reducing its toxic effects [58], [59].

The evidence for the association between konzo and cyanide comes essentially from ecological studies and no similar neurodamage secondary to exposure from other cyanide sources is known [57], [59]. These limitations need to be taken into account when considering cyanide toxicity as a cause of konzo.

The presence of high concentrations of unmetabolized linamarin in urine in individuals from konzo-affected communities compared with controls, and the potential of linamarin to induce neuronal lesions in experiments, support the possibility of linamarin as a cause of konzo [7], [50], [51].

AC, a metabolite of linamarin (Figure 2), is present in cassava flour consumed in association with konzo. As it is labile, its concentration decreases with time during storage. When an agricultural crisis results in food shortage, cassava may be consumed without previous storage, increasing exposure to AC. In experiments on rats, this metabolite caused selective neuronal degeneration in different brain areas, including non-cortical areas. Even considering the obvious limitations of an animal model, this finding suggests a possible contribution of this metabolite to konzo neurotoxicity and could help explain the additional non-motor neurological findings in konzo patients. Undernutrition favored the appearance of disease in this model [53].

The possibility of neurotoxicity by nitriles such as AC is attractive, as in theory it could unify the pathogenesis of konzo with two other familiar and similar toxico-nutritional neurological diseases: lathyrism, a non-progressive bilateral symmetric paraparesis associated with consumption of grass pea (*Lathyrus sativus*), and TAN, which has been associated with cassava consumption for many years. In theory, different nitriles present in cassava and grass pea could cause different direct neurotoxic patterns and/or different diseases [60].

Excitotoxic damage related to glutathione deficiency in konzo and lathyrism is also under debate [61]. SAAs such as methionine are essential for the synthesis of brain glutathione, which is the most abundant intracellular antioxidant and an important agent for detoxification of xenobiotics [62]. The absence of the cytoprotective glutathione, secondary to deficiency of SAA, would expose the brain to cyanogen neurotoxicity in konzo. Oxidative stress can be induced by exercise, and levels of glutathione transiently decrease during physical activity [63], [64]. As konzo often installs during or after exercise, it could be a consequence of an exacerbation of a chronic state of neuron glutathione deficiency.

Recently, thiamine deficiency, due to the use of thiamine sulphur for cyanide detoxification, was proposed as a possible cause of konzo [65]. Although peripheral neuropathy is sometimes reported in konzo epidemics, the absence of other typical manifestations of thiamine deficiency, such as Wernicke encephalopathy, Korsakoff syndrome, or wet beriberi, does not favor this hypothesis [66], [67].

Traditionally, thiocyanate is considered to be an innocuous product of cyanide detoxification, but some authors have proposed that it may have an aetiological role in konzo [59].

## From Individual Susceptibility to the Clinical Expression of Konzo

### Susceptibility to Konzo

People are not uniformly affected by konzo. Children above the age of 3 years and women in the fertile age group are more affected than adult males [1]–[8].

Many possible reasons can explain these differences. Adult males are more privileged in terms of finding and eating additional sources of food. Women and their accompanying children may be more exposed to cassava cyanide because they eat the toxic fresh roots, and may have additional exposure from cyanide inhalation during preparation or storage.

Pregnancy and breast-feeding are additional nutritional stresses that could predispose women to the toxic effects of cassava [9]. Biological differences, as in other neurological diseases, could affect susceptibility to cyanogens [68].

Breast-feeding children do not get konzo [1]–[8]. Studies have shown that the mammary gland barrier reduces thiocyanate passage from maternal serum to milk [69], [70]. We lack information about the presence in breast milk of the other candidates for neurotoxicity under the magnitude of exposure that occurs in konzo, and with the associated nutritional deficiency. Breast-fed children are, however, unlikely to be directly exposed to most of the potentially toxic compounds, as the milk has been processed in the body of the mother.

Familial clustering of cases of konzo is found in every reported outbreak. Such clustering does not discriminate between genetic susceptibility and environmental factors such as socioeconomic status and food intake. In the case of konzo, the age and gender distribution is more likely to be explained by common exposure, than genetic susceptibility.

### The Clinical Expression of Konzo

Acute intoxication symptoms due to cyanide include tachypnoea, tachycardia, dizziness, headache, abdominal pain, vomiting, diarrhea, mental confusion, and convulsions, generally 4–6 hours after ingestion of meals containing cassava [58], [71]–[73]. These symptoms are sometimes reported during konzo epidemics and in acute poisoning cases are attributed to cassava consumption, but without any recognizable motor sequelae [71]–[73].

**Figure 4:**
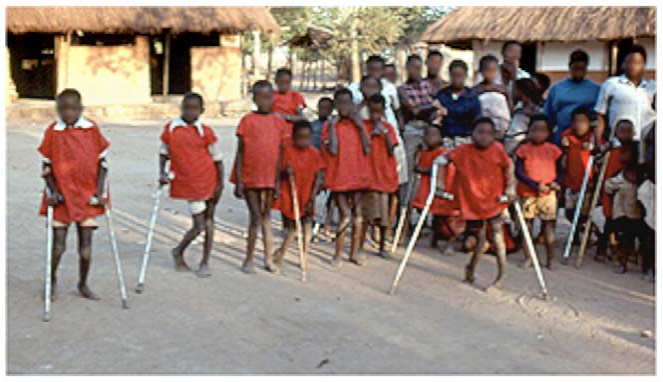
Children with konzo in a rural area of Mozambique (faces blurred).

The hallmark of konzo is difficulty in walking, and often the affected person walks with the help of sticks (Figure 4).

Usually, in less than a week, an acute and non-progressive symmetric spastic paraparesis becomes installed. The disease sometimes starts during or after manual work or a long walk. Although it starts abruptly, konzo follows the consumption of a monotonous diet based on cassava with high cyanide content for several weeks [6]–[10].

Transient non-motor symptoms such as paraesthesiae in the lower limbs, cramping, pain or trembling in the legs, and low back pain can precede or accompany the paraparesis of konzo, and disappear in weeks [2], [8]–[10].

Upper limb involvement in konzo varies from impairment of fine motor function accompanying spastic paraparesis, to a spastic tetraparesis, preventing autonomous ambulation [8], [9], [74]. Additional neurological findings such as optic neuropathy, rotatory nystagmus, pseudobulbar dysarthria, and hypoacusia are seen in some cases [6], [9], [23], [75], [76].

Signs of upper motor damage, in the form of spasticity resulting in exaggerated reflexes, ankle clonus, and extensor plantar reflexes are clinical markers in the absence of paraparesis. Studies have shown that the rate of ankle clonus is high in apparently healthy school children from affected areas 2 years after the epidemics [77].

**Figure 5:**
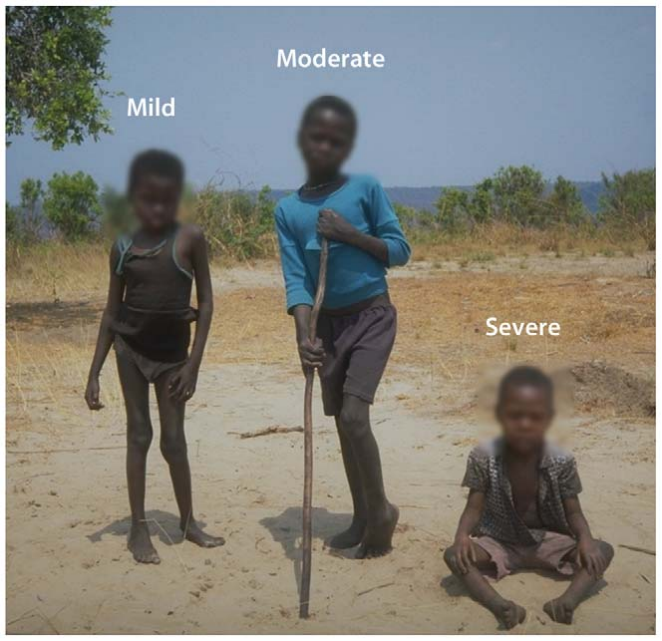
Different degrees of severity of konzo in children from Democratic Republic of Congo (faces blurred). Image Credit: Thorkild Tylleskar.

Depending on its severity, konzo is divided into three categories: mild when individuals are able to walk without support, moderate when individuals need one or two sticks to walk, and severe when the affected person is unable to walk (Figure 5) [10].

**Figure 6:**
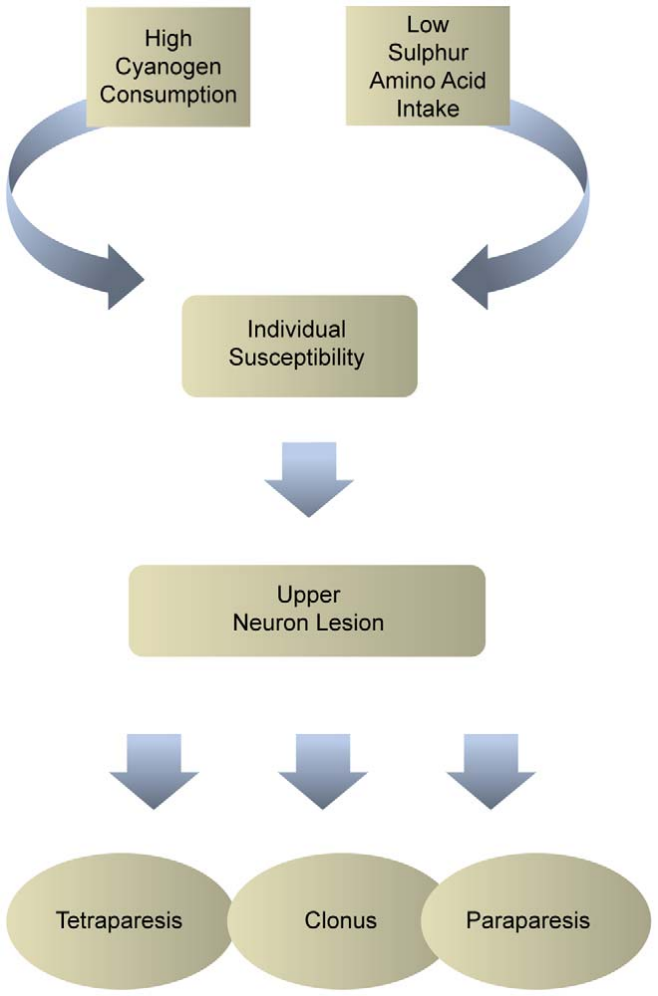
From individual susceptibility to the spectrum of konzo.

Using a comprehensive definition of disease, konzo should include the complete spectrum of clinical manifestations, ranging from “minor” isolated signs such as clonus to severe forms with tetraparesis (Figure 6). The case definition of konzo recommended by the World Health Organization (WHO) is based on the combination of visible spastic abnormalities in walking, a history of sudden onset in a formerly healthy person, and bilaterally exaggerated knee or ankle jerks without signs of disease of the spine [10]. By using these criteria, people with clonus and mild spasticity, but without visible difficulty in walking, are not considered to have konzo. However, because these minor signs are present in apparently healthy people [77], and they are only recognizable if neurological examination is performed, case detection and comparison between case series would be difficult to carry out.

On general examination, no consistent abnormality is found in konzo, but signs of malnutrition, anaemia, and splenomegaly may be present [76].

Second attacks have been reported in around 10% of patients. Contractures are a commonly developing complication over the years in more severe cases without physiotherapy, and painful calf muscle spasm may be a major chronic symptom [9], [74], [78].

There is no proven treatment for konzo. WHO recommends dietary diversification and immediate treatment with high doses of multivitamins, particularly vitamin B, in order to avoid increased neurodamage due to concurrent vitamin deficiency [10].

There is a lack of consistent studies assessing excess mortality in konzo, but in Tanzania and DRC, increased death rates were reported [2], [21].

## Conclusion and Future Directions

Taking Africa as a whole, konzo may not be a major public health problem, but for affected communities, the disease is a major burden. Increasing cassava production, declining production of other foods, global warming, more frequent droughts, wars, and population displacement have set the scene for konzo to persist.

Early recognition of konzo and active case detection is important to reveal the real extension of any konzo outbreak. Local authorities should promptly initiate interventions to avoid further cases.

Immediate interventions to prevent konzo in affected areas, such as providing food and vitamins, and promotion and dissemination of methods such as the wetting method to detoxify cassava flour in some affected areas, are essential.

In a long-term perspective, other interventions include improving food diversity and intake through more investment and support to rural agriculture. Introduction of new low cyanide cassava varieties that are also high yielding and disease resistant may also be considered.

Further research is needed on the impact of global warming on cassava production and cyanogen content. At a local level, determination of the acceptability on a wider scale of efficient processing methods is needed. Finally, the pathophysiology of konzo still needs to be elucidated.

**Key Learning Points**

- Konzo is a neurological entity found in Africa characterized by an abrupt onset of an irreversible, non-progressive, and symmetrical spastic para/tetraparesis. It affects the poorest people from the remotest areas in the continent.
- Poverty is the main risk factor, and commercialization, drought, or wars are the usual precipitators.
- The number of cases is probably underestimated; the disease continues to be reported and is expanding to new areas of the continent.
- The epidemics are associated with prolonged high dietary cyanogen consumption from insufficiently processed roots of bitter cassava combined with a protein-deficient diet low in sulphur amino acids.

**5 Key Papers in the Field**

- Cliff J, Lundquist P, Mårtensson J, Rosling H, Sörbo B (1985) Association of high cyanide and low sulphur intake in cassava-induced spastic paraparesis. Lancet 326: 1211–1213.
- Tylleskär T, Banea M, Bikangi N, Cooke RD, Poulter NH, Rosling H (1992) Cassava cyanogens and konzo, an upper motoneuron disease found in Africa. Lancet 15: 339–440.
- Tylleskär T, Howlett PW, Aquilonius SM, Stålberg E, Rosling H, et al. (1993) Konzo: a distinct disease entity with selective upper motor neuron damage. J Neurol Neurosurg Psychiatry 56: 638–643.
- Tshala-Katumbay D, Eeg-Olofsson K, Kazadi-Kayembe T, Tylleskär T, Fällmar P (2002) Analysis of motor pathway involvement in konzo using transcranial electrical and magnetic stimulation. Muscle Nerve 25: 230–235.
- Nhassico D, Muquingue H, Cliff J, Cumbana A , Bradbury JH (2008) Rising African cassava production, diseases due to high cyanide intake and control measures. J Sci Food Agric 88: 2043–2049.

## Supporting Information

Alternative Language Abstract S1 Translation of the abstract into Portuguese by the authors.
(0.03 MB DOC)
