# Supplementary material for: Konzo: From Poverty, Cassava, and Cyanogen Intake to Toxico-Nutritional Neurological Disease
Source: PLoS Negl Trop Dis. 2011 Jun 28;5(6):e1051. doi: 10.1371/journal.pntd.0001051 (PMC3125150; doi:10.1371/journal.pntd.0001051)
Supplement: Alternative Language Abstract S1 — Translation of the abstract into Portuguese by the authors. (0.03 MB DOC) [file pntd.0001051.s001.doc]

**Resumo**
Konzo é uma entidade neurológica distinta com lesão selectiva do neurónio motor superior,  caracterizada por um início súbito de para/tetraparésia espástica simétrica, irreversível, e  não progressiva. Apesar da sua gravidade, continua a ser uma doença negligenciada.   A doença está associada a um consumo elevado de glicosídeos cianogénicos provenientes da mandioca amarga em combinação com uma dieta deficiente em proteínas. As epidemias de konzo ocorrem quando estas duas condições coexistem em contexto de escassez grave de alimentos. Até 1993, as epidemias em áreas rurais pobres de África contribuíram com cerca de 3.700 casos de konzo.  O número de pessoas afectadas é subestimado. Relatos não oficiais apontam para um  número  estimado de pelo menos 100 mil casos no ano 2000, contrastando com os 6788 casos contabilizados até 2009 em  artigos publicados.
